# Prophylactic Left Atrial Appendage Ligation During Coronary Artery Bypass Graft Surgery Allows Safe Avoidance of Anticoagulation Regardless of Postoperative Atrial Fibrillation

**DOI:** 10.7759/cureus.59876

**Published:** 2024-05-08

**Authors:** Zain Khalpey, Usman Aslam, Parker Wilson, Jessa Deckwa, Ujjawal Kumar

**Affiliations:** 1 Department of Cardiothoracic Surgery, HonorHealth, Scottsdale, USA; 2 Department of General Surgery, HonorHealth, Phoenix, USA; 3 Department of Internal Medicine, Baylor University Medical Center, Dallas, USA; 4 Department of Research, Nihon Kohden Digital Health Solutions, Irvine, USA; 5 School of Clinical Medicine, University of Cambridge, Cambridge, GBR

**Keywords:** stroke, anticoagulation, coronary artery bypass grafting, postoperative atrial fibrillation, left atrial appendage ligation

## Abstract

Background

New-onset postoperative atrial fibrillation (POAF) is the most common arrhythmia following cardiac surgery. POAF increases the risk of thromboembolism and stroke, as well as morbidity and mortality more generally. Despite evidence from the landmark PROTECT-AF and PREVAIL trials, left atrial appendage ligation (LAAL) is not routinely performed for thromboembolism prophylaxis in POAF, and anticoagulation remains the standard of care along with dual antiplatelet therapy. This study evaluated the efficacy of concomitant LAAL in eliminating the need for postoperative anticoagulation, regardless of POAF development, in patients undergoing coronary artery bypass grafting (CABG).

Methods

Between 2019 and 2021, 130 patients were selected to undergo concomitant LAAL while undergoing CABG surgery. Patients were then monitored for the incidence of new-onset POAF, and anticoagulation was strictly avoided for this indication. Demographic and outcome data were collected, with endpoints including transient ischemic attack (TIA) or stroke, death, and readmission within one year, as well as the length of hospital and intensive care unit (ICU) admissions.

Results

POAF occurred in 37 patients (28.5%), consistent with previous reports. However, none of the POAF patients experienced TIA or stroke during the one-year follow-up period, compared to 2 (2.15%) in the non-POAF group, a typical rate of postoperative stroke in such a patient population. No significant differences were observed between POAF and non-POAF cohorts in one-year stroke, all-cause mortality, readmission rates, or total hospital stay. Interestingly, the POAF cohort had a significantly longer mean ICU stay (4.24 vs 3.37 days, p = 0.0219), possibly due to the time required for arrhythmia control before discharge. The study population had a high mean CHA_2_DS_2_-VASc score (2.81), indicating an increased risk of thromboembolism, and a high mean HAS-BLED score, suggesting an elevated bleeding risk with anticoagulation.

Conclusions

LAAL appears to be an effective adjunct to CABG for thromboembolism prophylaxis in POAF. Formal anticoagulation was avoided in this study, with no significant differences in adverse events between POAF and non-POAF groups, suggesting that LAAL may be a suitable alternative to anticoagulation, especially in high-risk patients (e.g., those with elevated CHA_2_DS_2_-VASc or HAS-BLED scores). The safety and efficacy of this approach should be corroborated by larger randomized studies, such as the ongoing LeAAPS trial. LAAL during CABG may help reduce the risk of bleeding complications associated with anticoagulation while maintaining protection against thromboembolic events in patients who develop POAF.

## Introduction

New-onset postoperative atrial fibrillation (POAF) is a common complication following cardiac surgery, occurring in an estimated 25% of patients [1]. The development of POAF is multifactorial, with inflammation, structural myocardial changes, and electrophysiological alterations in depolarization all playing a role [2,3]. Certain types of cardiac surgery, such as mitral and aortic valve repair, may further increase the risk of POAF [4]. The occurrence of POAF is associated with increased morbidity and mortality, including a greater risk of stroke, heart failure, prolonged hospital stays, and in-hospital mortality [5]. Fear of a postoperative stroke is a significant concern for many patients before opting to proceed with cardiac surgery. Sun and colleagues found that over 80% of patients surveyed would sacrifice longevity to avoid the risk of postoperative stroke [6]. As such, the identification and management of POAF are critical aspects of postoperative care.

Stroke is a particularly concerning thromboembolic consequence of POAF. Studies have shown that patients who develop POAF following cardiac surgery have a two-fold increase in the absolute risk of short-term stroke within 30 days of surgery compared to those who do not develop POAF [7]. AF patients have a four to five-fold increased risk of severe thromboembolic complications, including stroke, due to increased blood stasis [8,9]. Given this risk, anticoagulation is a standard component of postoperative treatment for cardiac surgery patients. However, the use of anticoagulants and antiplatelet agents carries an increased risk of hemorrhage and other side effects, especially in frail and elderly populations [10].

To mitigate these risks and potentially avoid the need for anticoagulation, several solutions have been proposed for thromboembolic prophylaxis following cardiac surgery. Many of these solutions focus on managing the left atrial appendage (LAA), as it is the most common site of left atrial thrombi in AF, with an estimated 90% of thrombi in nonvalvular AF originating from the LAA [11,12]. The LAA also plays a role in the development of AF due to its endocrine and barometric functions [13]. Therapies involving the left atrial appendage have consistently been targeted to reduce stroke risk, especially in cardiac surgery patients. While percutaneous endocardial LAA occlusion using the Watchman implant (Boston Scientific, Marlborough, MA) is effective in patients with nonvalvular AF in the PREVAIL and PROTECT-AF trials, surgical left atrial appendage ligation (LAAL) has also demonstrated promising results [14-16].

The multi-national randomized controlled LAAOS III trial showed that surgical ligation of the LAA with an epicardial clip, in addition to coronary artery bypass grafting (CABG), reduced the risk of ischemic stroke or systemic embolism compared to CABG alone in patients with a history of atrial fibrillation, even with similar rates of postoperative anticoagulation in both groups [17]. The ATLAS feasibility trial also demonstrated that LAAL reduced cerebral thromboembolism risk in patients at high risk for stroke and bleeding compared to no ligation [18]. Furthermore, Gerçek and colleagues showed the long-term durability of these results, with a 4.1% decrease in stroke incidence over five years following LAAL in addition to CABG compared to CABG alone, and a 6.3% stroke incidence reduction in patients with CHA_2_DS_2_-VASc scores > 3 [19]. These findings suggest that excluding the LAA presents a unique opportunity to address potential thromboembolism in the setting of POAF, potentially eliminating the need for anticoagulation in patients with no prior history of atrial fibrillation.

In this study, we evaluated the efficacy of LAAL in patients undergoing CABG to eliminate the need for postoperative anticoagulation, regardless of the development of POAF. Patient outcomes and adverse events were examined for up to one year postoperatively to assess the efficacy and safety of LAA closure. Findings from this study were presented in part at the Society of Thoracic Surgeons’ Coronary Conference in Ottawa on the 4th and 5th of June 2022.

## Materials and methods

Study design and patient population

This retrospective, non-randomized cohort study included 130 consecutive patients with normal sinus rhythm who underwent CABG surgery performed by a single surgeon between 2019 and 2021 at Northwest Medical Center in Tucson. The study was approved by the Institutional Review Board (IRB#20200195), and informed consent for the surgical procedures and anonymized inclusion into the study was obtained from all patients. The study was conducted in accordance with the relevant guidelines and regulations for working with human subjects.

Inclusion criteria were an age of at least 18 years and angiographically confirmed coronary artery disease warranting revascularization. A multidisciplinary team, including cardiac surgeons and interventional cardiologists, selected all patients for revascularization via CABG rather than percutaneous approaches. Exclusion criteria included a clinical history of atrial fibrillation or any pre-existing diagnoses of liver impairment, chronic kidney disease (CKD), or coagulopathy. Patients undergoing concomitant surgical AF ablation or any other cardiac surgical procedure to reduce atrial arrhythmias were also excluded.

Heart failure was defined using American Heart Association (AHA) criteria for diastolic heart failure as an ejection fraction of less than or equal to 40% by transthoracic echocardiography [20]. CKD was defined using the Kidney Disease: Improving Global Outcomes (KDIGO) guidelines, which define CKD as a glomerular filtration rate < 60 ml/min/1.73m^2^ and albuminuria with an albumin-creatinine ratio > 3 mg/mmol [21].

Data collection

Demographic data, preoperative characteristics, and postoperative outcomes were collected from the electronic health record system (Epic Systems Corporation, Madison, USA) [22]. The collected data were anonymized and stored on a secure server in accordance with institutional information governance protocols for outcome research. Preoperative characteristics included comorbidities such as chronic lung disease, congestive heart failure, hypertension, prior myocardial infarction (MI), cerebrovascular accidents (CVAs), transient ischemic attack (TIA), pulmonary embolism (PE), or peripheral embolism.

The primary outcome endpoints were the occurrence of a radiologically confirmed CVA or TIA within one year, one-year all-cause mortality, and hospital readmission within one year. The secondary endpoints were the length of stay (LOS) in the intensive care unit (ICU) and total hospital admission. Patients were evaluated for the development of new-onset POAF during the immediate postoperative admission following CABG surgery to segregate the study population into two cohorts for suitable comparison.

Operative procedure

**Figure 1:**
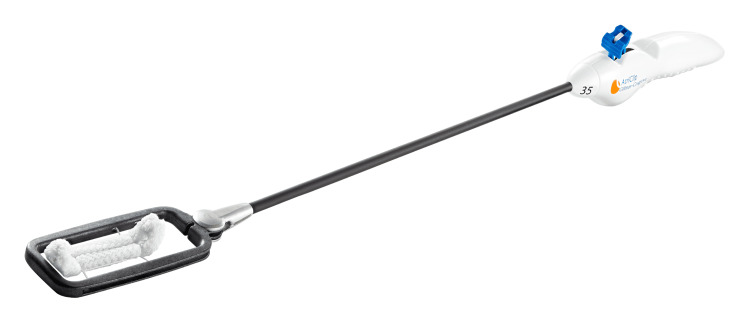
The AtriClip® delivery device and pre-loaded left atrial appendage clip. The 35 mm model is shown here, featuring a malleable 25 cm shaft, end effector with 180 degrees total head articulation, and thumb lever deployment. Image used with permission of AtriCure, Inc., Mason, OH, USA.

All patients underwent successful, uncomplicated CABG with adjunctive left atrial appendage ligation using a 40 mm AtriClip (AtriCure, Mason, OH, Figure 1). In all cases, the clip was applied while the aortic was still cross-clamped to reduce the risk of any embolization to the brain. The grafts are placed behind the graft to prevent impinging the graft once the clip is placed and deployed. Typically, in our practice, the left internal mammary artery (LIMA) is used to bypass the left anterior descending (LAD) coronary artery, with the remaining vessels being bypassed using saphenous vein grafts, which will occasionally be carried out sequentially. The radial artery is very rarely used, and the right internal mammary artery is not used in our practice. Patients received no formal postoperative anticoagulation, only standard-of-care dual antiplatelet therapy (DAPT) following surgery. All patients provided full informed consent for enrollment in the study and were adequately informed of the risks and benefits of the procedures and the avoidance of anticoagulation.

Postoperative follow-up

Following CABG surgery, all patients were initiated on standard DAPT: daily aspirin (81 mg) for life and clopidogrel (75 mg) for one year to maintain graft patency, as per standard institutional practice, even for patients who did not undergo LAAL. Patients did not receive any anticoagulant therapy at any time, regardless of POAF occurrence. POAF was diagnosed according to the Society of Thoracic Surgeons (STS) criteria for input into the national Adult Cardiac Surgery database [23]. These stipulate that the atrial fibrillation or flutter episode should last for at least 30 seconds. The episode should occur during the postoperative period, after cardiac surgery, and before hospital discharge. These criteria specify that atrial fibrillation or flutter should be documented by a 12-lead electrocardiogram (EKG), rhythm strip, or continuous telemetry monitoring. Patients with a history of preoperative atrial fibrillation or flutter were excluded unless it was successfully treated with ablation or surgery, and they had consistently been in normal sinus rhythm preoperatively. Lastly, reversible causes of atrial fibrillation, such as electrolyte imbalances or hyperthyroidism, should be ruled out or treated before making a diagnosis of postoperative atrial fibrillation.

Patients were followed up for one year, and no anticoagulation was initiated during this period. The outcomes evaluated included all-cause mortality, TIA, CVAs within one year, hospital readmissions within one year, and total hospital and ICU length of stay (LOS). Outpatient clinic follow-up occurred at two weeks, four weeks, six months, and one year postoperatively, where a full clinical history and examination were undertaken, including specific questions regarding neurological symptoms to evaluate the study endpoints. The occurrence of a stroke was defined by a permanent residual neurological deficit with radiologically confirmed changes.

Statistical analysis

Data were summarized using descriptive statistics. For continuous variables, mean and standard deviation (SD) were presented for normally distributed data. Cohort differences in continuous variables were compared using t-tests, and categorical variables were compared using Fisher's exact test between POAF and non-POAF patients. All statistical analyses were performed using GraphPad Prism version 10.1.0 for macOS (GraphPad Software, Boston, MA, USA) [24], and a p-value < 0.05 was considered significant, as is conventional.

## Results

**Figure 2:**
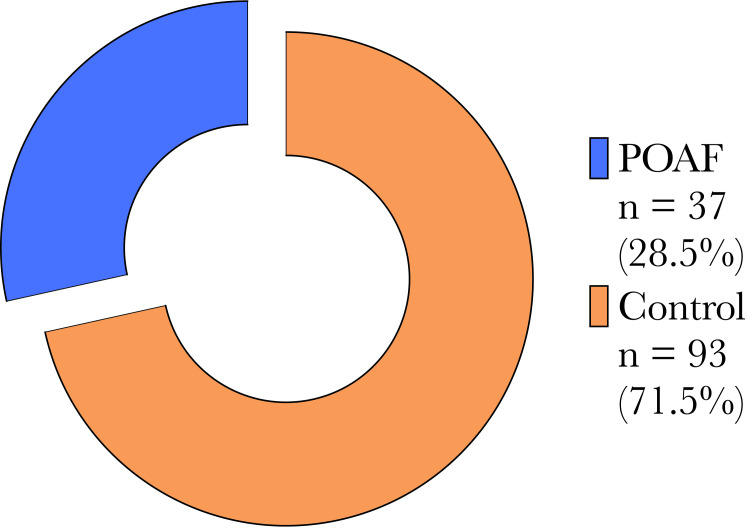
Cohort distribution segregated by the development of postoperative atrial fibrillation (POAF). Distribution of patients within the study who developed new-onset postoperative atrial fibrillation (n = 37), compared to the control cohort who remained in normal sinus rhythm postoperatively (n = 93).

A total of 130 patients undergoing concomitant LAAL during CABG were included in this study. LAAL was achieved without complication in all patients. The AtriClip did not interfere with any grafts, and there were no cases of kinking or impingement. Thirty-seven patients (28.5%) developed POAF during their immediate postoperative admission, as shown in Figure 2.

Full demographic data with statistical analysis and calculated p-values are presented in Table 1.

**Table 1:** Baseline characteristics stratified by POAF occurrence. Demographics and risk factors of patients included in this study, segregated by the development of POAF. Categorical variables are expressed as N (%), with continuous variables expressed as mean ± SEM. POAF: Postoperative atrial fibrillation; MI: myocardial infarction; CVA: cerebrovascular accident

Variable	POAF (n = 37)	Control (n = 93)	p-value
Age, y	70 ± 7.86	66 ± 9.27	0.0242
BMI, kg/m^2^	28.97 ± 5.44	28.77 ± 4.85	0.8383
CHA_2_DS_2_-VASc	2.86 ± 1.48	2.78 ± 1.41	0.7744
HAS-BLED	2.46 ± 1.24	2.08 ± 1.02	0.0759
STS Score	1.25 ± 1.03	1.50 ± 1.28	0.2940
Sex, Male	29 (78.38%)	70 (75.27%)	0.8214
CHA_2_DS_2_-VASc ≥ 2	32 (86.49%)	74 (79.57%)	0.4568
HAS-BLED ≥ 2	30 (81.08%)	66 (70.97%)	0.2753
Congestive Heart Failure	5 (13.51%)	9 (9.68%)	0.5392
Hypertension	27 (72.97%)	75 (80.65%)	0.3517
Chronic Lung Disease	2 (5.41%)	19 (20.43%)	0.0372
Prior MI	12 (32.43%)	21 (22.58%)	0.2687
Prior CVA	4 (10.81%)	5 (5.38%)	0.2733
Prior Pulmonary Embolism	1 (2.7%)	2 (2.15%)	> 0.9999
Prior Peripheral Embolism	2 (5.41%)	5 (5.38%)	> 0.9999

**Figure 3:**
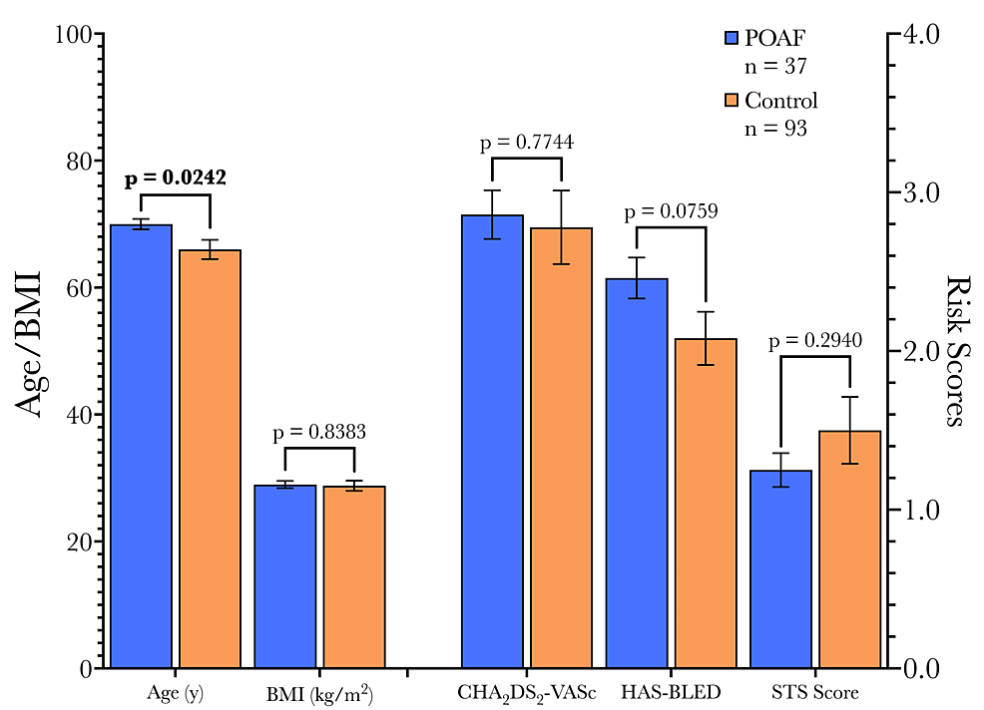
Demographic variables and risk scores. The POAF cohort was significantly older than the control cohort. Otherwise, groups were similar. Cohort differences in continuous variables were compared using t-tests, and categorical variables were compared using Fisher's exact test between POAF and non-POAF patients. POAF: Postoperative atrial fibrillation

The POAF cohort was significantly older than the control group (70 years vs. 66 years), consistent with age being an established risk factor for atrial fibrillation [25]. Other preoperative characteristics, including body mass index (BMI), CHA_2_DS_2_-VASc score, HAS-BLED score, and STS risk score, were similar in the two cohorts (Figure 3). Beta-blocker (BB) use was similar between groups.

Interestingly, chronic lung disease was significantly less common in patients who developed POAF (20.43% vs. 5.41%), contradicting the consensus that chronic lung disease is associated with atrial fibrillation due to chronic hypoxia-mediated atrial remodeling [26]. There were no other significant differences in past medical history between the two cohorts. A full outcome comparison between the POAF and control cohorts is shown in Table 2.

**Table 2:** Outcomes stratified by POAF occurrence. Outcomes as compared between cohorts. Categorical variables are expressed as N (%), with continuous variables expressed as mean ± SEM. Cohort differences in continuous variables were compared using t-tests, and categorical variables were compared using Fisher's exact test between POAF and non-POAF patients. N/A: Not applicable; LOS: length of stay; POAF: postoperative atrial fibrillation

Variable	POAF (n = 37)	Control (n = 93)	p-value
Stroke	0 (0%)	2 (2.15%)	> 0.9999
Death	0 (0%)	0 (0%)	N/A
Readmission	3 (8.11%)	10 (10.75%)	0.7572
Hospital LOS	6.700 ± 3.70	5.900 ± 3.29	0.2297
ICU LOS	4.240 ± 1.95	3.370 ± 1.92	0.0219

**Figure 4:**
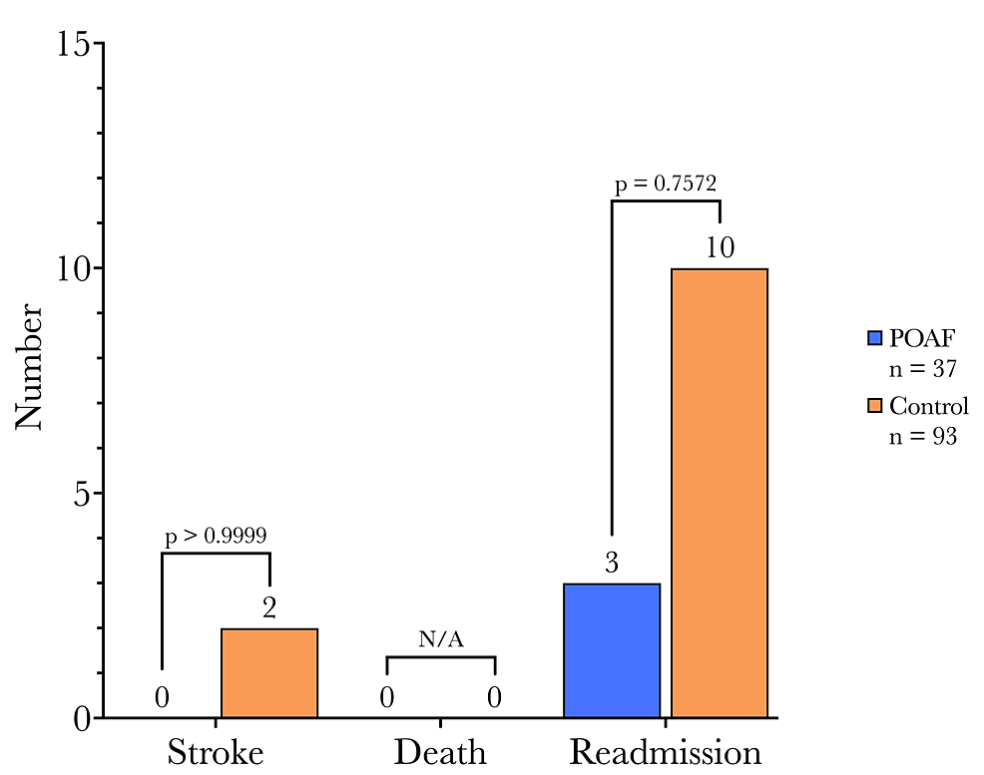
Primary endpoints of stroke, death, or readmission within one year: groups had similar outcomes. Primary endpoints: No significant differences were observed at one year between the two cohorts. Cohort differences in continuous variables were compared using t-tests, and categorical variables were compared using Fisher's exact test between POAF and non-POAF patients. N/A: Not applicable; POAF: postoperative atrial fibrillation

Regarding the primary endpoints of stroke, death, or readmission within one year, there were no significant differences between the two cohorts (Figure 4). Within the one-year follow-up period, none of the patients who developed POAF experienced a TIA or ischemic CVA. However, two patients (2.15%) who remained in normal sinus rhythm postoperatively developed a TIA or CVA in the same period. Of the patients who experienced POAF, three (8.12%) were readmitted within one year compared to 10 (10.75%) of the control cohort. None of the patients in either cohort of this study died during our investigation.

**Figure 5:**
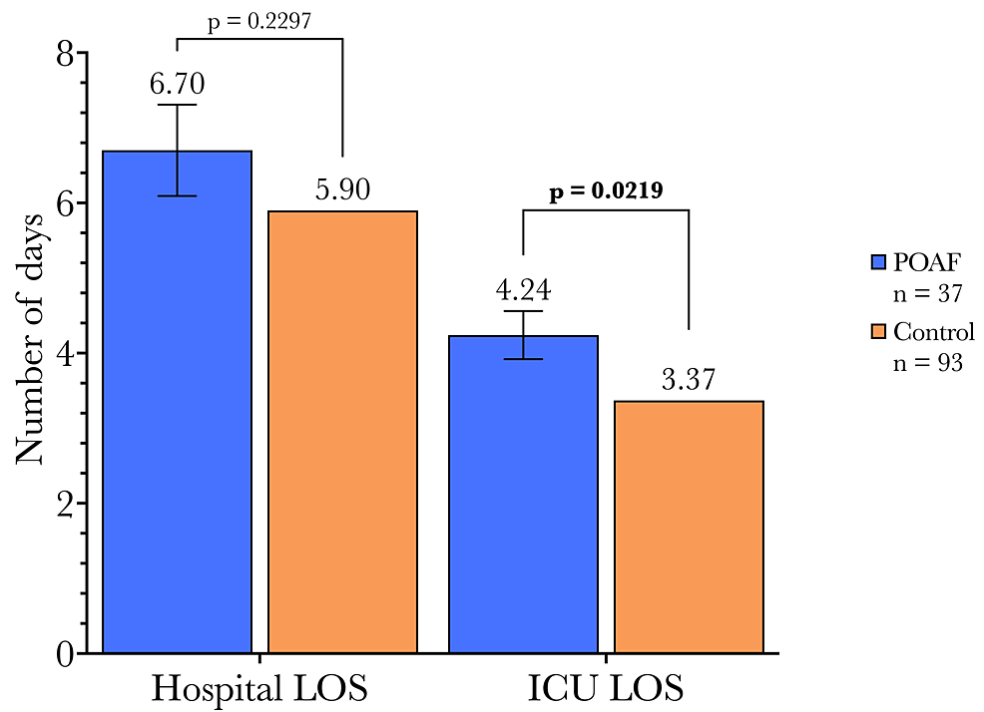
Postoperative length of stay and ICU stay stratified by POAF occurrence. Secondary endpoints: There was a significantly longer length of ICU admission in the POAF cohort than in the Control cohort, the total length of hospital admission was not significantly different. Cohort differences in continuous variables were compared using t-tests, and categorical variables were compared using Fisher's exact test between POAF and non-POAF patients. LOS: Length of stay; ICU: intensive care unit; POAF: postoperative atrial fibrillation

Overall, postoperative LOS was not significantly different between POAF and control cohorts (6.70 days vs. 5.90 days). However, there was a significant difference in the length of ICU admission, with POAF patients having longer stays compared to those without an atrial fibrillation event (4.24 days vs. 3.37 days), as shown in Figure 5.

## Discussion

The primary objective of this study is to demonstrate that concomitant LAAL during CABG surgery renders formal anticoagulation therapy, in addition to the standard-of-care DAPT for six months, redundant. Our findings have shown that the elimination of formal anticoagulation is a safe approach following CABG, even in the event of new-onset POAF. This was achieved by investigating the impact of LAAL on the incidence of postoperative thromboembolic events (cerebral ischemia leading to neurological deficits), hospital LOS, readmission rates, and mortality over a one-year follow-up period.

Of the patients who developed POAF following CABG and LAAL, none experienced a CVA and TIA or exhibited symptoms of neurological deficits. Conversely, 2 out of 93 patients (2.15%) who remained in normal sinus rhythm did experience either a TIA or definitive CVA over the subsequent year. This observed rate of thromboembolic events is consistent with the typical stroke rates of 1-3% following coronary surgery, as reported in the literature [27]. Upon further review of the postoperative progress notes for these two patients, it was revealed that they had presented with neurological symptoms within 12 hours of surgery, had received large quantities of packed red blood cell blood products, and had a preoperative left ventricular ejection fraction of <35%, necessitating postoperative inotropic support. Blood transfusion has previously been shown to be an independent predictor of stroke after cardiac surgery [28]. These findings suggest an alternative mechanism for neurological deficits other than ischemia secondary to cerebral thromboembolism, most likely insufficient intraoperative cerebral perfusion.

These data, therefore, indicate that LAAL was successful in reducing the risk of thromboembolism without the need for formal anticoagulation in the setting of POAF. The low incidence of CVA or TIA (2/130, 1.54%) observed in both cohorts may be attributed, at least in part, to the LAAL procedure, and we believe this provides further promising evidence of the utility of LAAL as a prophylactic measure against thromboembolism.

This finding is particularly significant considering that our patient population was at an elevated risk for thromboembolism. The CHA_2_DS_2_-VASc score is a validated system used to stratify the one-year risk of thromboembolism in nonanticoagulated patients with nonvalvular atrial fibrillation. In our study population, the mean CHA_2_DS_2_-VASc score for all patients was 2.81 ± 1.42, and over 80% had scores ≥ 2, which is the typical threshold for considering oral anticoagulation therapy. Furthermore, each patient within our study had at least one of the following risk factors for thrombus formation in the LAA: left atrial appendage velocity ≤ 40cm/s, left atrial size ≥ 5.5cm, or CHA_2_DS_2_-VASc score ≥ 2. These factors are strong predictors of LAA thrombus formation and, consequently, the risk of future embolic events such as stroke [29,30].

Additionally, the patient population within our study was also shown to have increased risks of adverse bleeding events with anticoagulation therapy. The HAS-BLED score is a validated tool for estimating an individual patient's risk of major hemorrhage if placed on anticoagulation therapy, thereby assessing the quality of atrial fibrillation care. Many of our patients had HAS-BLED scores ≥ 2, implying that they would be at a high risk of bleeding if placed on formal anticoagulation, which would typically have formed part of the management strategy should these patients have developed POAF. Therefore, the avoidance of anticoagulation in this study may have prevented devastating hemorrhagic consequences, including gastrointestinal or intracerebral bleeding. This is a primary reason to avoid anticoagulation, and if LAAL is effective in reducing the risk of thromboembolism, it lends credence to the consideration of prophylactic LAAL as an alternative to postoperative anticoagulation.

Overall, 28.5% (37/130) of the patients enrolled in this study developed POAF, which is a similar incidence to past reports and studies [31]. It does not appear that LAAL reduces the incidence of POAF in the setting of CABG. Although patients who experienced POAF had longer stays in the ICU (4.24d vs. 3.37d, p = 0.0219), their total hospital LOS was not significantly longer (6.70d vs. 5.90d, p = 0.2297). This could be accounted for by the need to manage POAF and increased vigilance in the ICU setting due to the potential for vital sign instability. However, it is important to note that this arrhythmia did not delay hospital discharge, as we showed the length of hospital admission to be similar between groups. This result is in relative contrast to typical POAF patients who do not receive adjunctive LAAL, who typically remain in the hospital for longer periods than patients who do not develop POAF. This is often due to thromboembolic events that occur due to POAF, hence why this is not seen in our study population.

Such patients typically require prolonged hospital stays (an additional 2 to 5 days) and incur an average of $10,000 - $20,000 in additional treatment costs [4,32,33]. Our data indicates that adjunctive LAAL could, therefore, potentially save healthcare resources and costs by decreasing hospital stays for patients with new-onset POAF. Regarding a cost-benefit analysis for adjunctive LAAL, forthcoming work from our institution has evaluated the financial implications. According to this analysis, the estimated cost per AtriClip device utilized for LAAL is approximately $1800, which we feel is justified given the potentially greater hospitalization costs if POAF were to develop. There was no left atrial appendage intervention as discussed.

Over the year-long follow-up period, there were fewer readmissions for patients who developed POAF than for those who remained in sinus rhythm, although the difference was not statistically significant. Overall, our outcomes data suggest that since LAAL did not lengthen hospital stays or increase readmission rates, this procedure is safe, and its efficacy has already been demonstrated, as discussed.

It is important to recognize the limitations of our study. First, this study was conducted at a single center, with patients undergoing surgery by a single surgeon. This limitation is being actively addressed by our center's involvement in the LeAAPS trial (NCT05478304) [34]. This is a prospective, randomized controlled trial currently underway to evaluate prophylactic stroke reduction via routine LAAL, aiming to show prognostic benefit to concomitant LAAL. An additional limitation of this study is that the follow-up period was only one year, so a key element of our ongoing work is reassessing these 130 patients at future time points, for example, at two and five years postoperatively.

Ultimately, the management and prevention of POAF is a complex endeavor that requires a multidisciplinary approach from a preoperative, intraoperative, and postoperative perspective. Preoperatively, it is important to note that one of the major risk factors for POAF is age, as each decade of increasing age is associated with a 75% increase in the odds of developing POAF, placing patients who are more than 70 years of age at a much greater risk [35]. Due to structural age-related changes, coronary artery disease may be more advanced in older patients, further increasing the risk of POAF. Postoperatively, beta-blockers have been consistently shown to be useful prophylactic and treatment options for POAF, reducing morbidity and mortality [36]. Other antiarrhythmic drugs, such as amiodarone, have also been used to curb morbidity in POAF patients [37]. Intraoperative POAF prophylaxis and treatment, however, are not well-detailed in the literature. We believe that LAAL should be considered as part of the intraoperative cardiothoracic surgical armamentarium for preventing thromboembolism in the event of POAF.

## Conclusions

This study provides compelling evidence that concomitant LAAL during CABG may eliminate the need for formal anticoagulation in patients who develop POAF while still providing adequate thromboembolism prophylaxis. None of the POAF patients who underwent LAAL experienced a stroke or TIA, despite having elevated CHA_2_DS_2_-VASc scores indicating high thromboembolic risk. Furthermore, LAAL did not increase total hospital length of stay or readmission rates compared to those who remained in normal sinus rhythm postoperatively. These findings suggest that LAAL is a safe and effective adjunct to CABG, particularly in high-risk patients where the avoidance of anticoagulation could prevent devastating bleeding complications. While larger randomized trials are still needed to corroborate these results, such as the ongoing LeAAPS trial, this study highlights the potential utility of LAAL as an alternative to postoperative anticoagulation for thromboembolism prophylaxis in POAF patients undergoing cardiac surgery.
